# 

**Published:** 1882-11

**Authors:** 


### Pamphlets.

---

Tubucular tumors of the windpipe. Tuberculosis of the Laryngeal muscles. A contribution to the Pathological Histology of Laryngo-Tracheal Phthisis. By John N. Mackenzie, M.D., Baltimore. Reprint from the *Archives of Medicine.*

Alcoholic Anæsthesia. By Lewis D. Mason, M.D., Baltimore. Reprint from the *Journal of Inebriety.*

On the Continuous Inhalation of the vapor of slacking lime in the treatment of Membranous Laryngitis. By Eugene F. Cordell, M.D. Reprint from the *Maryland Medical Journal.*

Congenital Paralysis of the Sixth and Seventh Pairs of Cranial Nerves in an adult.

Cataract. Extraction with Iridectomy in an Infant six months old. By Julian J. Chisolm, M.D. Baltimore. Reprint from the *Archives of Ophthalmolgy.*

Stricture of the Rectum, treated by Electrolysis. By Robert Newman, M.D. New York. Reprint from *New England Medical Monthly.*

The First Bi-Ennial Report uf the Michigan Free Eye and Ear Infirmary, and Eye and Ear Department of the Michigan College Hospital. For the two years ending September 31, 1882. Compliments of Dr. C. J. Lundy.

---

It is asserted that carbolic acid, in the proportion of one part to one thousand, will keep solutions of atropia and eserine clear, and prevent the formation within them of a fungous growth, to the septic action of which the conjunctival inflammation, which is sometimes set up by the use of old atropia solutions, has been ascribed. Carbolic acid in this proportion produces no disagreeable sensation in the eye, and it is much more efficient than either salicylic or boracic acid, which have been used for the same purpose.

